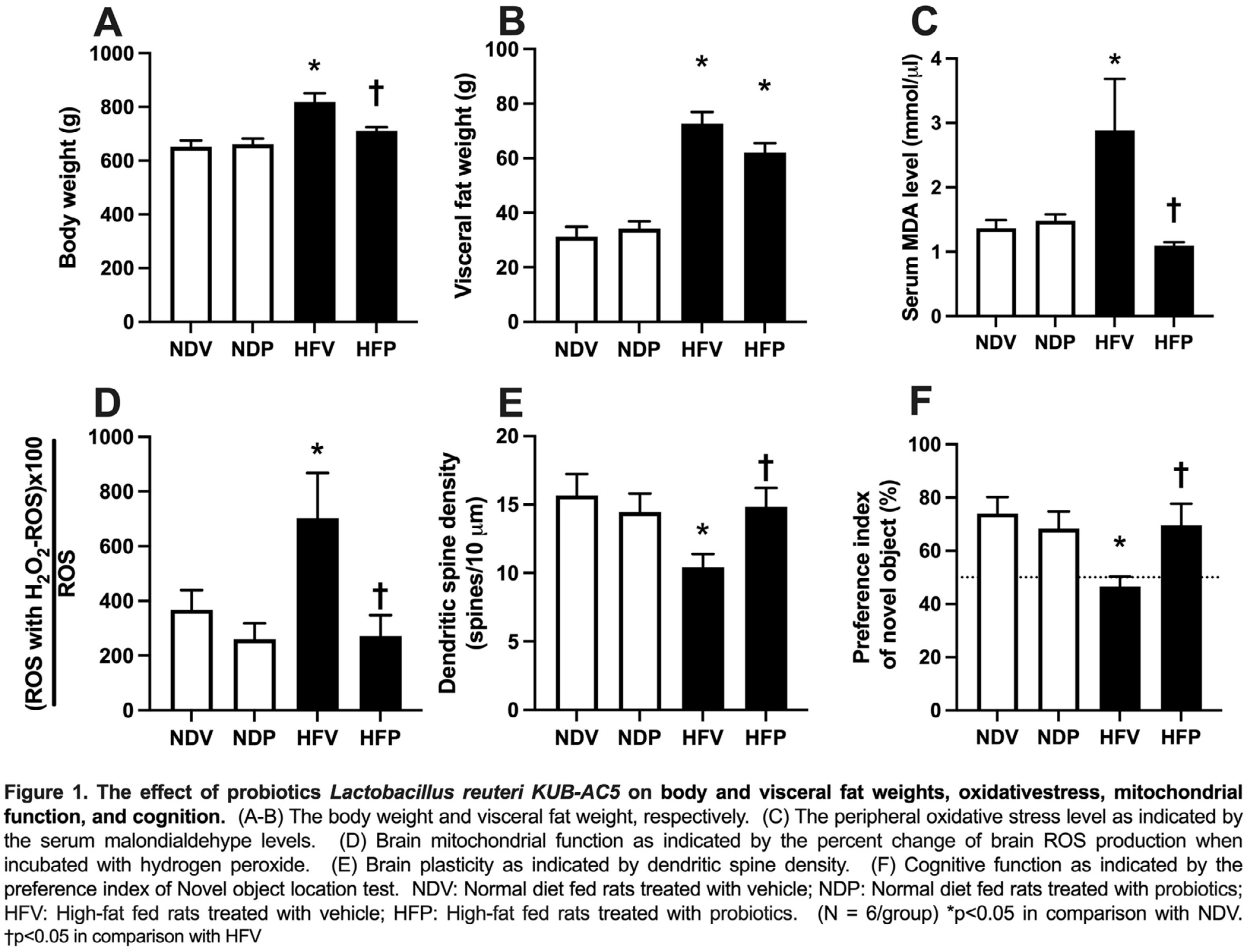# Probiotics *Lactobacillus reuteri KUB‐AC5* exerts neuroprotective effects on brain pathologies and attenuates depressive‐like behavior in obese insulin‐resistant rats

**DOI:** 10.1002/alz70855_098546

**Published:** 2025-12-23

**Authors:** Titikorn Chunchai, Hiranya Pintana, Patcharapong Pantiya, Busarin Arunsak, Suriphan Donchada, Chanon Kunasol, Sasiwan Kerdphoo, Wichwara Nawara, Chanisa Thonusin, Parameth Thiennimitr, Nipon Chattipakorn, Siriporn C Chattipakorn

**Affiliations:** ^1^ Neurophysiology Unit, Cardiac Electrophysiology Research and Training Center, Faculty of Medicine, Chiang Mai University, Chiang Mai, Thailand; ^2^ Center of Excellence in Cardiac Electrophysiology Research, Chiang Mai University, Chiang Mai, Thailand; ^3^ Cardiac Electrophysiology Unit, Department of Physiology, Faculty of Medicine, Chiang Mai University, Chiang Mai, Thailand; ^4^ Department of Microbiology, Faculty of Medicine, Chiang Mai University, Chiang Mai, Thailand; ^5^ Department of Oral Biology and Diagnostic Sciences, Faculty of Dentistry, Chiang Mai University, Chiang Mai, Thailand

## Abstract

**Background:**

Chronic consumption of a high‐fat diet (HFD) alters gut microbiota, induces endotoxemia, and impairs cognitive function (1, 2). Previous studies have shown that the administration of probiotics, such as *Lactobacillus paracasei* HII01, attenuated gut dysbiosis, alleviated metabolic disturbance, improved brain mitochondrial function, and improved cognitive function in obese insulin‐resistant rats. However, the effects of probiotics *lactobacillus reuteri KUB‐AC5* on these parameters in obese insulin‐resistant rats have not been investigated.

**Method:**

Twenty‐four male Wistar rats were fed either a normal diet (ND) or an HFD (59.28% energy from fat) for total 24 weeks. At week 13, each dietary group was then divided into subgroups receiving either vehicle or *L. reuteri* KUB‐AC5 (1 ml daily oral feeding of 10^8^ CFU) for an additional 12 weeks. At the end of the experimental protocol, the cognitive function was determined in all rats. Then animals were euthanized, and blood and brains were collected for further analysis.

**Result:**

Chronic HFD consumption increased body weight, visceral fat weight, total cholesterol, insulin levels, increased HOMA‐IR index, and increased serum malondialdehyde (MDA) level, when compared to ND‐fed rats treated with vehicle. HFD‐fed rats treated with probiotics significantly decreased body weight, insulin, serum MDA levels, and HOMA‐IR index, when compared to HFD‐fed rats treated with vehicle (*p* <0.05, Figure 1A‐C). In addition, the impairment of brain mitochondrial function was observed in HFD‐fed rats treated with vehicle by increasing percent change of brain ROS production when incubated with hydrogen peroxide, which was decreased in HFD‐fed rats treated with probiotics (*p* <0.05, Figure 1D). Moreover, HFD‐fed rats treated with vehicle decreased dendritic spine density and exhibited cognitive decline, which were attenuated in HFD‐fed rats treated with probiotics (*p* <0.05, Figure 1E‐F). We also observed that ND‐fed rats treated with probiotics did not alter all parameters.

**Conclusion:**

Our findings suggest that the administration of *L. reuteri* KUB‐AC5 mitigates cognitive decline in obese insulin‐resistant rats by alleviating metabolic disturbances, reducing peripheral oxidative stress, improving brain mitochondrial function, and improving neuronal plasticity. These findings highlight its potential as a therapeutic intervention for metabolic and neurodegenerative disorders.